# Draft Genome Sequence of *Salmonella enterica* subsp. *enterica* Serovar Infantis Strain SPE101, Isolated from a Chronic Human Infection

**DOI:** 10.1128/genomeA.00679-17

**Published:** 2017-07-20

**Authors:** Andrés Iriarte, Joaquín Giner-Lamia, Claudia Silva, Laura Betancor, Lizeth Astocondor, Juan J. Cestero, Theresa Ochoa, Coralith García, José L. Puente, José A. Chabalgoity, The SalmoIber CYTED Network, Francisco García-del Portillo

**Affiliations:** a Instituto de Higiene, Facultad de Medicina, Universidad de la República, Montevideo, Uruguay; b Laboratorio de Patógenos Bacterianos Intracelulares, Centro Nacional de Biotecnología, Consejo Superior de Investigaciones Científicas (CNB-CSIC), Madrid, Spain; c Departamento de Microbiología Molecular, Instituto de Biotecnología, Universidad Nacional Autónoma de México, Cuernavaca, Morelos, Mexico; d Instituto de Medicina Tropical Alexander Von Humboldt, Universidad Peruana Cayetano Heredia, Lima, Peru

## Abstract

We report a 4.99-Mb draft genome sequence of *Salmonella enterica* subsp. *enterica* serovar Infantis strain SPE101, isolated from feces of a 5-month-old breast-fed female showing diarrhea associated with severe dehydration and malnutrition. The infection prolonged for 6 months despite antibiotic treatment.

## GENOME ANNOUNCEMENT

*Salmonella enterica* subsp. *enterica* serovar Infantis is a nontyphoid emerging serovar showing increased morbidity in humans worldwide. Recent studies have shown that some emergent *S*. Infantis isolates carry a self-transmissible megaplasmid that confers stress tolerance and promotes pathogenicity, which can be horizontally transferred to the gut microbiota by conjugation (1, 2). *S*. Infantis plasmids are thus responsible for the dissemination of genes encoding extended spectrum beta-lactamases and quinolone resistance determinants (3). Surveillance studies also reveal an increased presence of *S*. Infantis in poultry meat (3), as well as prevalence in human persistent infections caused by nontyphoidal *Salmonella* serovars (4).

*S*. Infantis strain SPE101 was isolated from the feces of a 5-month-old breast-fed female in a pediatric unit of a public hospital in Lima, Peru, 4 months after initial hospitalization. The patient showed signs of dehydration and malnutrition when hospitalized. The *Salmonella* infection persisted for 6 months despite several regimens of beta-lactams, quinolone, and trimethoprim-sulfamethoxazole antibiotic treatments. Prior to genome sequencing, the SPE101 isolate was typified as *S*. Infantis by multiplex PCR (O and H antigens) and determined to belong to sequence type 32 (ST32) by multilocus sequence typing. The plasmid profile of this isolate revealed the presence of an ~250-kb megaplasmid, similar in size to the megaplasmid pESI reported in emergent *S*. Infantis isolates (1). PCR assays demonstrated the presence of the pESI backbone gene *traC* (5). Antimicrobial susceptibility tests performed according to the Clinical and Laboratory Standards Institute (6) showed resistance to beta-lactams and quinolones.

Genomic DNA from an overnight culture of SPE101 was obtained using the phenol-chloroform extraction method (7). Paired-end (2 × 150) sequencing was performed using the Illumina MiSeq platform (Illumina, Inc, San Diego, CA, USA). A total of 6,145,442 reads were generated. *De novo* assembly was conducted using an algorithm based on the de Brujin graphs (8), resulting in 63 contigs ≥ 500 bp with a 168-fold average coverage. The annotation was performed with the RAST algorithm (9). The draft genome of SPE101 contains 4,985,409 bp, with a 52.1% G+C content, and has 4,805 protein coding sequences, 11 rRNAs, 75 tRNAs, and 201 pseudogenes.

The 4,805 predicted SPE101 proteins were searched using tBLASTn in the 75 genomes of *S*. Infantis isolates deposited in GenBank by April 2017. Seven proteins were not encoded by any other available genome of *S*. Infantis. Among them were two putative transcriptional regulators, a proline-rich protein, and proteins encoded by a lambdoid phage. Interestingly, 266 of the 286 genes annotated in the pESI megaplasmid of the emergent *S*. Infantis strain 119944 reported in Israel (1) were found in SPE101.

Further analyses with derivative strains of SPE101 lacking the genes absent in other *S*. Infantis isolates may provide the foundation toward unraveling novel attributes of this emergent serovar in human salmonellosis.

### Accession number(s).

This draft genome project has been deposited at DDBJ/ENA/GenBank under the accession number NBAV00000000 (BioProject PRJNA380335; BioSample SAMN06640695). The version described in this paper is the first version, NBAV01000000.
